# Machine learning identifies exosome features related to hepatocellular carcinoma

**DOI:** 10.3389/fcell.2022.1020415

**Published:** 2022-09-19

**Authors:** Kai Zhu, Qiqi Tao, Jiatao Yan, Zhichao Lang, Xinmiao Li, Yifei Li, Congcong Fan, Zhengping Yu

**Affiliations:** ^1^ Department of Hepatobiliary Surgery, The First Affiliated Hospital of Wenzhou Medical University, Wenzhou, China; ^2^ Wenzhou Business College, Wenzhou, China

**Keywords:** TCGA database, exosome, PI3K-Akt pathway, immune cell, machine learning, prognosis signature, HCC

## Abstract

**Background:** Hepatocellular carcinoma (HCC) is one of the most malignant tumors with a poor prognosis. There is still a lack of effective biomarkers to predict its prognosis. Exosomes participate in intercellular communication and play an important role in the development and progression of cancers.

**Methods:** In this study, two machine learning methods (univariate feature selection and random forest (RF) algorithm) were used to select 13 exosome-related genes (ERGs) and construct an ERG signature. Based on the ERG signature score and ERG signature-related pathway score, a novel RF signature was generated. The expression of BSG and SFN, members of 13 ERGs, was examined using real-time quantitative polymerase chain reaction and immunohistochemistry. Finally, the effects of the inhibition of BSG and SFN on cell proliferation were examined using the cell counting kit-8 (CCK-8) assays.

**Results:** The ERG signature had a good predictive performance, and the ERG score was determined as an independent predictor of HCC overall survival. Our RF signature showed an excellent prognostic ability with the area under the curve (AUC) of 0.845 at 1 year, 0.811 at 2 years, and 0.801 at 3 years in TCGA, which was better than the ERG signature. Notably, the RF signature had a good performance in the prediction of HCC prognosis in patients with the high exosome score and high NK score. Enhanced BSG and SFN levels were found in HCC tissues compared with adjacent normal tissues. The inhibition of BSG and SFN suppressed cell proliferation in Huh7 cells.

**Conclusion:** The RF signature can accurately predict prognosis of HCC patients and has potential clinical value.

## Introduction

Hepatocellular carcinoma (HCC) is the major histologic subtype of liver cancer, accounting for 90% of primary liver cancer. Currently, HCC is the third leading cause of cancer-related death worldwide, with the increasing incidence (Sung et al., 2021). HCC is mainly caused by viral infection, carcinogen, and chronic inflammation (Schulze et al., 2016). Despite improvements in systemic therapy, surgical technique, and radiation therapy, the overall 5-year survival rate of HCC patients is currently less than 20% (Fujiwara et al., 2018). Therefore, identifying prognostic markers for HCC is important to prolong patient survival time.

Exosomes are small extracellular nanovesicles with diameters of 30–150 nm that play an important role in cellular communication and epigenetic regulation by transporting key proteins and genetic material (e.g., miRNA, mRNA, and DNA) (Yang et al., 2020). Cancer cell-secreted exosomes, which are involved in cancer cell genesis and development, may promote tumor proliferation and metastasis by participating in cellular communication, regulating cell signaling, and promoting the formation of a pre-metastatic niche (Kharaziha et al., 2012; Hoshino et al., 2015; Peinado et al., 2017; Wortzel et al., 2019; Zhang and Yu, 2019). It has been shown that exosomes from cancer cells regulate antitumor immune response by inhibiting T-cell activation and proliferation, inducing regulatory T cells and bone marrow-derived suppressor cells, and suppressing natural killer (NK) and CD8^+^ T-cell functions (Wieckowski et al., 2009; Pyzer et al., 2016; Whiteside, 2016). Exosomal protein markers as well as nucleic acid markers can be used for early diagnosis and assessment of the prognostic risk of recurrence in a variety of cancers (Mashouri et al., 2019; Li et al., 2021). For example, the levels of exosomal CD151, CD171, and TSPAN8 have been proven to be effective indicators for predicting the prognosis of lung cancer (Iqbal et al., 2019). Nucleic acids such as miR-21 and miR-1246 in exosomes can be used as markers for breast cancer detection (Dong et al., 2020). Although the predictive significance of exosomes in HCC has been investigated, there are no reports on the systematic detection of exosomal biomarkers in patients with HCC.

In this study, we used two machine learning methods (univariate feature selection and random forest (RF) algorithm) to screen out 13 exosome-related genes (ERGs) and constructed an ERG signature with significant prognostic prediction performance. Then, enrichment analysis identified some remarkable factors that correlated with the prognosis of HCC patients. Patients with both high-risk exosome scores and high-risk NK scores had the worst survival. Finally, ERG, mitosis, PI3K-Akt pathway, B cell, NK cell, and CD8^+^ T cell were integrated into an RF signature by an RF algorithm. This RF signature was shown to have a better prognostic prediction performance than any single factor. Then, real-time quantitative polymerase chain reaction (PCR) and immunohistochemistry (IHC) were performed to verify the differential expressions of prognostic genes in HCC tissues and adjacent normal tissues.

## Materials and methods

### Data collection

The patient data from TCGA LIHC (https://portal.gdc.cancer.gov/projects/TCGA-LIHC) included 424 cases. The samples included 374 tumor samples and 50 normal samples. Samples from the International Cancer Genome Consortium (ICGC) were downloaded at https://dcc.icgc.org/releases/current/Projects/LIRI-JP. Among them, RNA-seq data were available for 231 cases, of which 9 patients presented multiple duplicates of tumor samples. ERGs were obtained at http://www.exocarta.org/, with a total of 2700 genes (Supplementary Table S1). The obtaining of 30 pairs of HCC and adjacent tumor tissue samples from The First Affiliated Hospital of Wenzhou Medical University (FAHWMU) was approved by the FAHWMU Ethics Committee. Moreover, written informed consent was also obtained from the participating patients.

### Gene selection and construction of the ERG signature

We first used univariate feature selection to identify the best genes correlated with the overall survival (OS) time using Python 3.9.7. Univariate feature selection is a simple method for evaluating the importance of features using univariate statistical tests. Then, we built a classifier using RF algorithm, and genes with feature importance >0.06 were selected (Breiman, 2004). RF is a classifier that contains multiple decision trees. Its output category is determined by the mode of the category of the output of the individual tree. Univariate Cox regression analysis was used to identify whether those selected genes are independent prognostic factors (*p* <0.05). Least absolute shrinkage and selection operator (LASSO) was then used for further selection. The optimum penalty parameter (λ) for the model was determined by ten-fold cross-validation following the minimum criteria (i.e., the value of λ corresponding to the lowest partial likelihood deviance). The correlation coefficient was calculated using the Spearman correlation analysis. The finally selected genes in TCGA cohort included: LDHA, PRDX1, HADHA, SFN, UBB, S100A10, BSG, LRP4, HSPA8, CALM1, PON1, IDH1, and PRDX6.

ERG scores of the patients were calculated according to the normalized expression of each gene and its corresponding coefficient from the feature importance calculated by RF algorithm. The formula was established as follows:

$$ERG\;score=e^{sum\left(expression\;level\;of\;each\;gene\times corresponding\;coefficient\right)}.$$

#### Functional enrichment

Patients were stratified into high- or low-risk groups based on the median value of their ERG score. Then, we screened different expression genes (DEGs) between low- and high-risk groups by the “limma” package of R (|log2FC| ≥ 1, FDR <0.05). Gene Ontology (GO) and Kyoto Encyclopedia of Genes and Genomes (KEGG) analyses for DEGs were performed using the OmicShare tools (https://www.omicshare.com/tools). The protein–protein interaction (PPI) networks of DEGs were generated by the STRING database (STRING: functional protein association networks (string-db.org)).

The “prcomp” function of the “stats” R package was used to perform principal component analysis (PCA). Additionally, using the “Rtsne” R package, we explored the clustering of different groups using t-distributed stochastic neighbor embedding (t-SNE). In order to determine the predictive accuracy of the model, the “pROC” R package was used to analyze receiver operating characteristic (ROC) curves. The survival analysis was performed by the Kaplan–Meier method. Using CIBERSOFT algorithm, the relative content score of 22 tumor immune-infiltrated cells in each HCC patient was calculated. The heatmap was drawn using the “pheatmap” package of R.

### Gene normalization

The genes used for calculating the mitosis score, NK score, PI3K-Akt score, B cell score, and CD8^+^ T score were downloaded from GSEA (https://www.gsea-msigdb.org/gsea/msigdb/index.jsp) or previous literature works (Bolotin et al., 2017; Hydbring et al., 2017; Mariathasan et al., 2018; Dyugay et al., 2022) (Supplementary Table S1). All those genes were normalized by LASSO and univariate Cox regression to improve the accuracy of prognosis prediction (Supplementary Table S2).

### Construction of the machine learning signature

Random forest modeling is one of the most universal machine-learning algorithms, which can model response prediction via fitting training data on the basis of different input features. It was performed using the Random Forest Classifier from the Python scikit-learn library. The hyperparameters of the model (i.e., the maximal amount of samples in the leaf and tree depth) were optimized with the use of RandomizedSearchCV and GridSearchCV from the Python scikit-learn library with 5-fold cross-validation. The F1 score was used as a measure of quality to train the model:

$$F1=2\cdot \frac{precision\cdot recall}{precision+recall}.$$

The variance of the RF model can be explained as the difference between training and test set quality metrics.

To explore input feature importance and interactions, the RF model was applied to all patients with Shapley additive explanation (SHAP) (Lundberg et al., 2020). SHAP is a game theory method that interprets the machine-learning model and understands the decision-making process by quantifying the contribution that each feature brings to the prediction made by the model.

### Quantitative real-time PCR analysis

We performed different analyses for the 13 ERGs between HCC samples and normal liver samples (Supplementary Table S3). BSG, S100A10, and SFN had the smallest FDR value. In addition, BSG and SFN were shown to have a significant prognosis for HCC patients (Supplementary Figure S1). In this case, we chose BGS and SFN for further analysis.

We collected 15 pairs of HCC and adjacent tumor tissue samples from the FAHWMU. Total RNA was isolated from tumor tissues as well as adjacent normal tissues using the Tiangen RNA extraction reagent kit. Each sample was reversely transcribed into complementary DNA (cDNA) using a reverse-transcription (RT) reagent kit (Takara Biotechnology Co., Ltd., Dalian, China). Then, real-time PCR was performed using SYBR Premix ExTaq (Takara). GAPDH was used as an endogenous control for mRNAs.

### Immunohistochemistry

We collected 15 pairs of HCC and adjacent tumor tissue samples from the FAHWMU. The tissues were immersed in 10% formalin for fixation, and then the formalin-fixed tissues were degreased and rehydrated. Next, the sections, blocked in 10% BSA, were incubated with anti-SFN and anti-BSG primary antibodies at 4°C for at least 12 h. Then, the sections were incubated with a horseradish peroxidase-conjugated secondary antibody for 30 min. Finally, quantitative analysis for SFN- and BSG-positive areas was performed under the microscope (Carl Zeiss, Germany).

### Cell culture

The human HCC cell line Huh7 was purchased from ATCC. Huh7 was cultured in DMEM with 10% fetal bovine serum (FBS) and 1% antibiotics. The cells were maintained in a 37°C incubator with 5% CO_2_.

### Cell transfection

The Huh7 cell line was cultured in a six-well plate at a density of 8 × 10^3^ cells per well. When the cell density was near to 50%, si-NC, si-SFN, and si-BSG packaged by lipo2000 were transfected into cells at 37°C for 6 h. Then, the fresh medium was replaced, and the cells were collected for subsequent experiments after 48 h of transfection.

### Cell proliferation assay

Cell counting kit-8 (CCK-8) (Dojindo, Japan) was used for the assessment of cell proliferation. Cells were seeded into a 24-well plate at a density of 1 × 10^5^/100 ml per well and incubated for 48 h. Then, 100 μl CCK-8 solution was added to each well and maintained in a 37°C incubator for 1 h. Finally, the absorbance of each well was measured at 450 nm.

### Statistical analysis

R software (version 4.0.3) and Python software (version 3.9.7) were used to complete all statistical work and plot drawings. Survival plots were created using the Kaplan–Meier estimator. The AUC was calculated with the “pROC” R package. For comparing multiple survival curves, univariate Cox regression analysis was used. The relative reliability of the RF signature was estimated by the Akaike information criterion (Akaike, 1974) and concordance index (Lin, 1989). Differences between two groups were compared using Student’s t-test. *p* < 0.05 was considered significant.

## Results

### Construction and verification of the ERG signature

**FIGURE 1:**
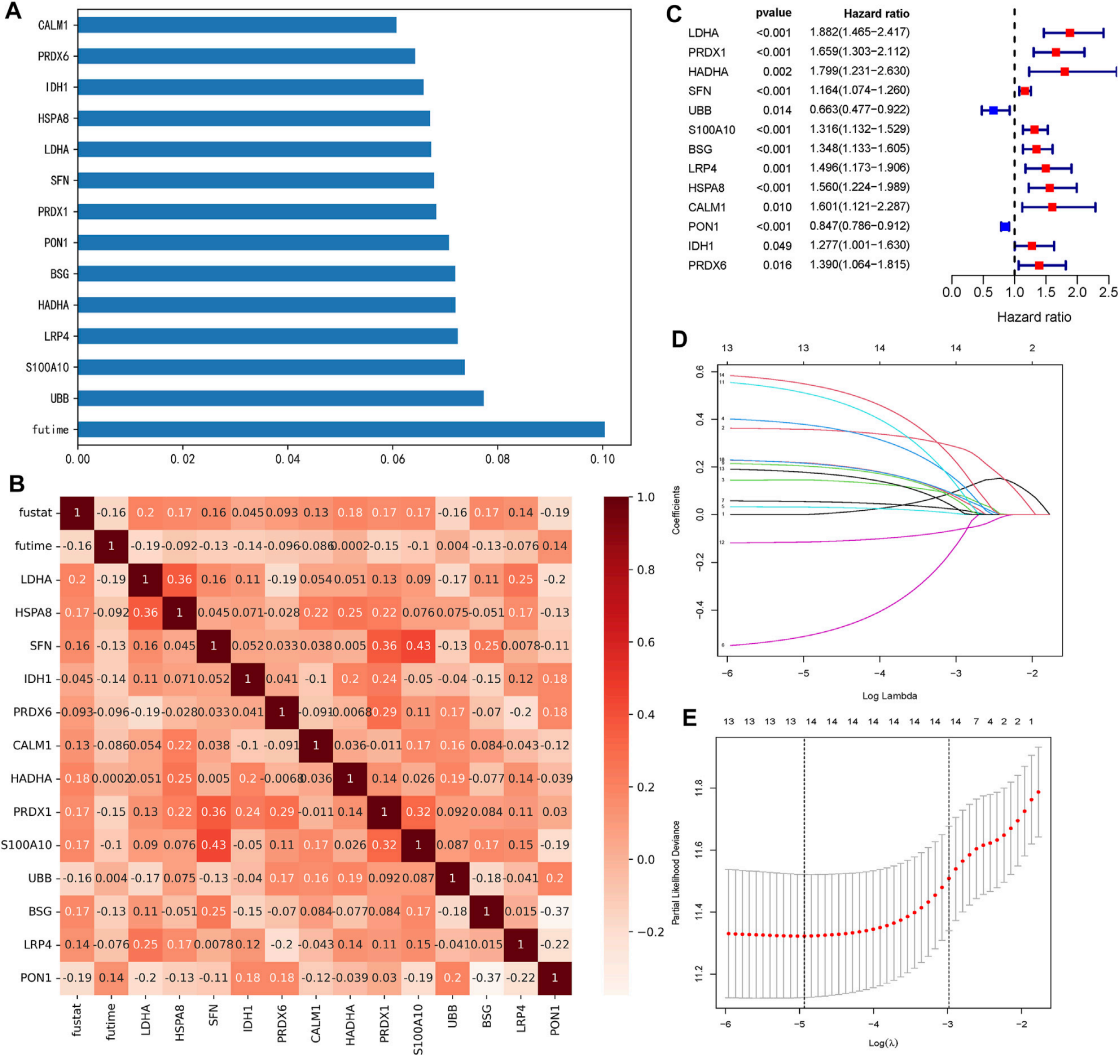
Selection of 13 ERGs. **(A)** Feature importance of 13 ERGs (each gene >0.06). **(B)** Heatmap of relationships among 13 ERGs with the survival time (futime) and survival state (fustat). Genes with the redder pattern color mean the greater correlation with the survival time and state. **(C)** Forest plots show the results of the univariate Cox regression analysis of 13 ERGs (*p* <0.05). **(D)** Ten-fold cross-validation for tuning parameter selection in the LASSO model. The solid vertical lines represent partial likelihood deviance ±standard error (SE) values. **(E)** LASSO coefficient profiles for the 13 DEGs.

From 2700 ERGs, we selected 13 genes with feature importance >0.06 (Figure 1A). The Spearman correlation coefficients among 13 ERGs are shown in Figure 1B. Moreover, the relationships between 13 ERGs and OS time as well as the survival status were also analyzed (Figure 1B). Clearly, all these ERGs were significantly correlated with the survival status. The results of the univariate Cox regression analysis indicated that all 13 ERGs were independent prognostic factors for HCC (*p* <0.05). These ERGs passed LASSO selection (lambda.min = −4.9 and lambda.1se = −3) (Figures 1C,D). The ERG score was calculated using mRNA expression levels and relevant coefficients of these 13 ERGs.

**FIGURE 2:**
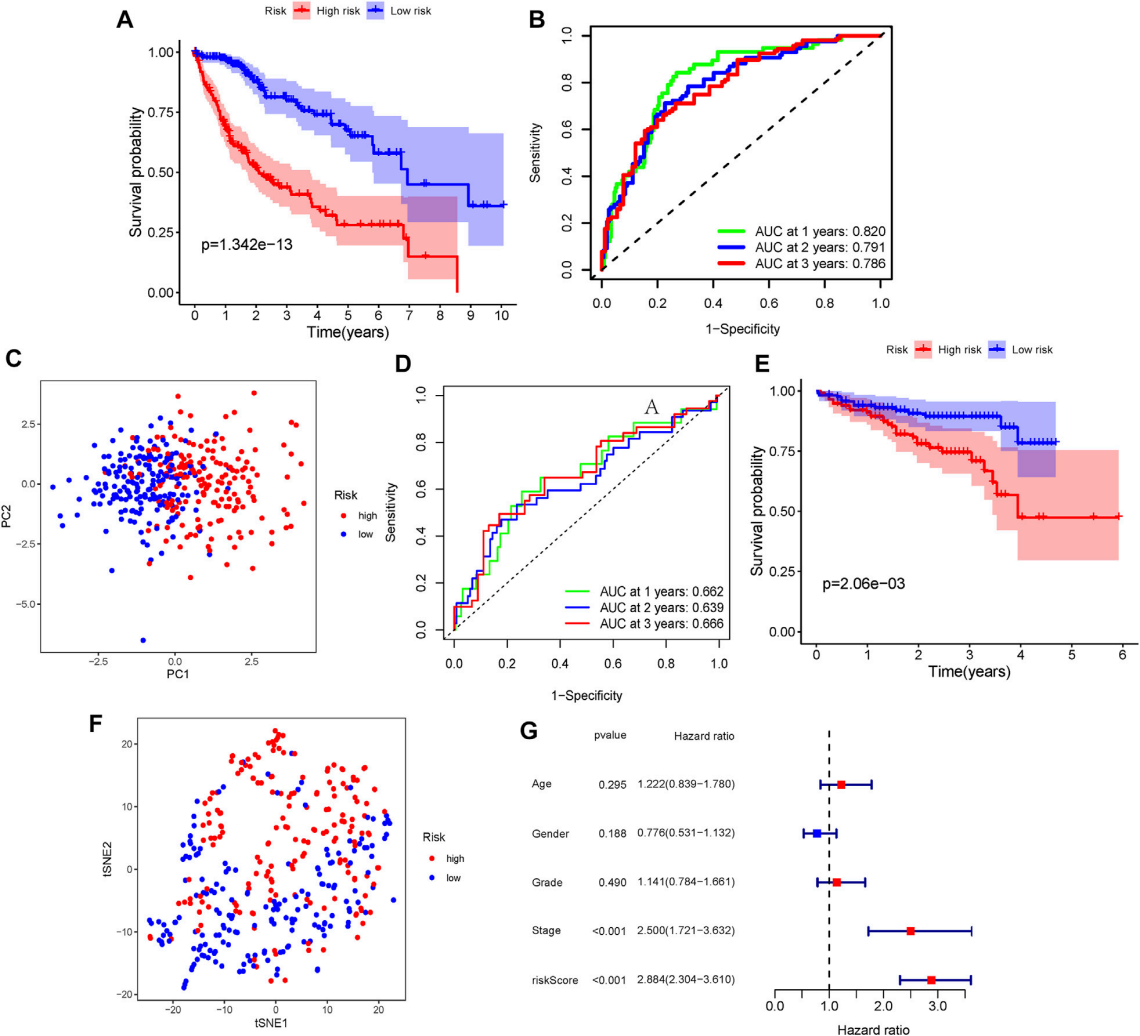
Verification of the ERG signature. **(A)** Kaplan–Meier curves for the OS of patients between the high-risk group and low-risk group (divided by the ERG signature) in TCGA cohort. **(B)** AUC of time-dependent ROC curves verified the prognostic performance of the risk score in TCGA cohort. **(D)** Kaplan–Meier curves in the ICGC cohort. **(E)** AUC of time-dependent ROC curves in the ICGC cohort. **(C,F)** t-SNE and PCA dimension reduction analysis of the high- and low-risk groups based on the risk score in TCGA cohort. **(G)** Forest plots of univariate Cox regression analyses containing the clinical feature and risk score of the ERG signature in TCGA cohort.

Patients in TCGA and ICGC cohorts were then divided into the high- or low-risk groups according to the median cut-off value, respectively. The results of the Kaplan–Meier curve indicated that patients in the low-risk group exhibited a significantly better OS than those in the high-risk group in TCGA (Figure 2A, *p* = 1.342 × 10^−13^) and ICGC cohorts (Figure 2E, *p* = 0.0206). The predictive performance of the ERG score for OS was evaluated by time-dependent ROC curves. In TCGA cohort, the area under the curve (AUC) reached 0.820 at 1 year, 0.791 at 2 years, and 0.786 at 3 years (Figure 2B). In the ICGC cohort, the AUC was 0.662 at 1 year, 0.639 at 2 years, and 0.666 at 3 years (Figure 2D). The PCA and t-SNE plots showed that the ERG score can distinguish the low-risk group from the high-risk group in TCGA cohort (Figures 2C,F). The result of the univariate Cox regression analysis indicated that the ERG score and stage of patients were independent prognostic factors (Figure 2G, *p* <0.001).

### Identification of the mitosis score

**FIGURE 3:**
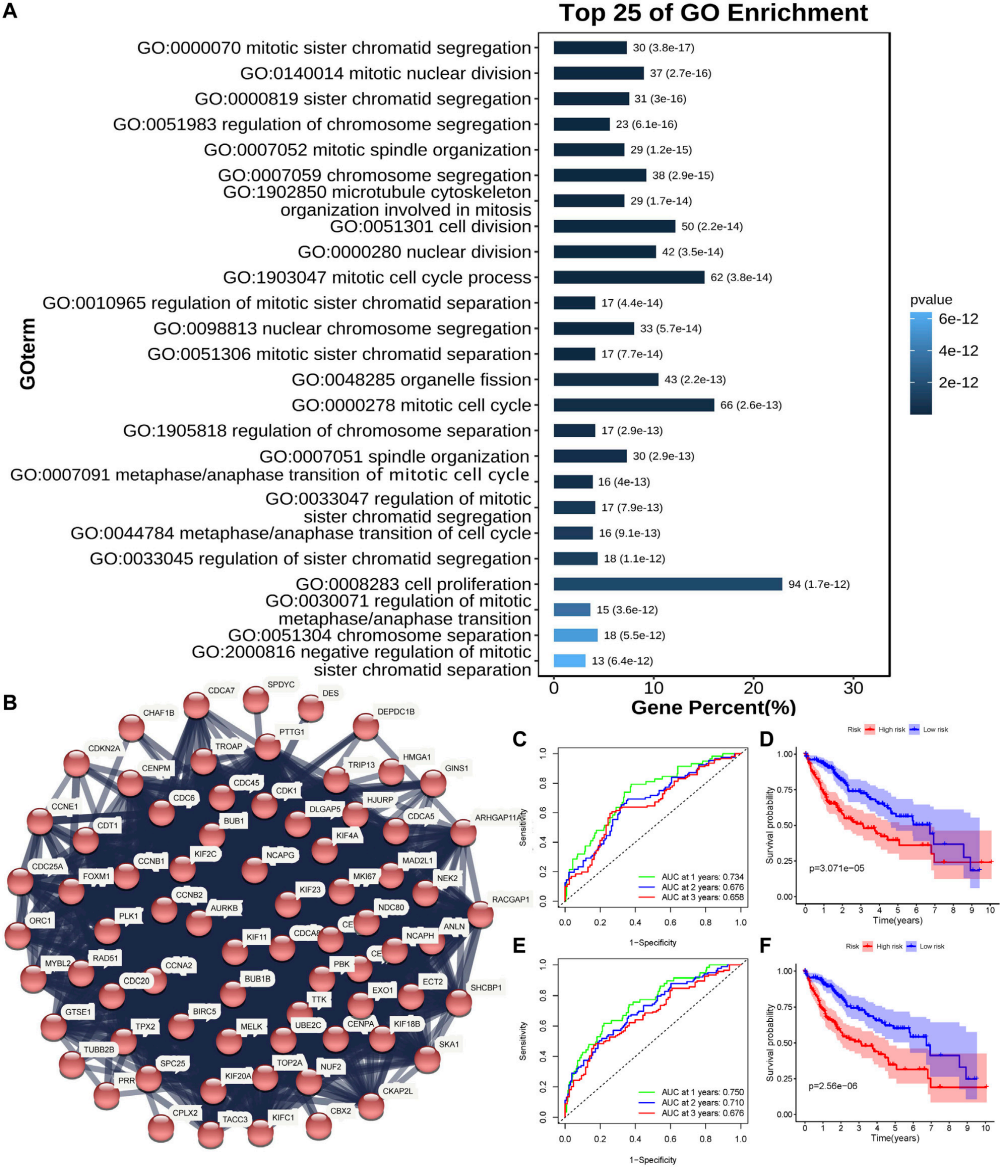
Identification of the mitosis score. **(A)** Top 25 enriched genes identified using GO enrichment analysis of DEGs between high- and low-risk groups (*p* < 0.05, *q* < 0.05). **(B)** PPI network constructed using the STRING database illustrated interactions among the DEGs. The Markov clustering algorithm identified the biggest cluster. **(C,D)** AUC of time-dependent ROC curves **(C)** and Kaplan–Meier curves **(D)** of the non-normalized mitosis-related gene signature in TCGA. **(E,F)** AUC of time-dependent ROC curves **(E)** and Kaplan–Meier curves **(F)** of the normalized mitosis-related gene signature in TCGA.

DEGs were subsequently identified in the high- and low-risk groups. As presented in Figure 3A, GO analysis showed that the differential genes were mainly enriched in mitotic-related processes (mitotic sister chromatid segregation, mitotic nuclear division, sister chromatid segregation, and cell proliferation, etc.). The associations between these DEGs were analyzed and clustered through the STRING website. The largest cluster was mainly enriched in mitotic-related genes (Figure 3B). Full cluster genes are listed in Supplementary Table S4.

A study has proposed a mitosis-related gene signature for tumors (Hydbring et al., 2017). Genes for the mitosis-related gene signature are shown in Supplementary Table S1. Then, we applied this signature to HCC patients in TCGA cohort. The Kaplan-Meier curve showed that a lower score of the signature was associated with better HCC OS (Figure 3D, *p* < 0.05). The AUC was 0.734 at 1 year, 0.676 at 2 years, and 0.658 at 3 years (Figure 3C). Then, we used univariate Cox regression and LASSO analyses to normalize mitosis-related genes (BRSK1, CDKN2B, GML, KIF2C, RAN, and TTK). The mitosis signature had a better performance in predicting HCC prognosis after normalizing (Figure 3F, *p* < 0.05). Also, the AUC reached 0.750 at 1 year, 0.710 at 2 years, and 0.676 at 3 years (Figure 3E), which was better than the non-normalized signature.

### Immune-related processes were selected

**FIGURE 4:**
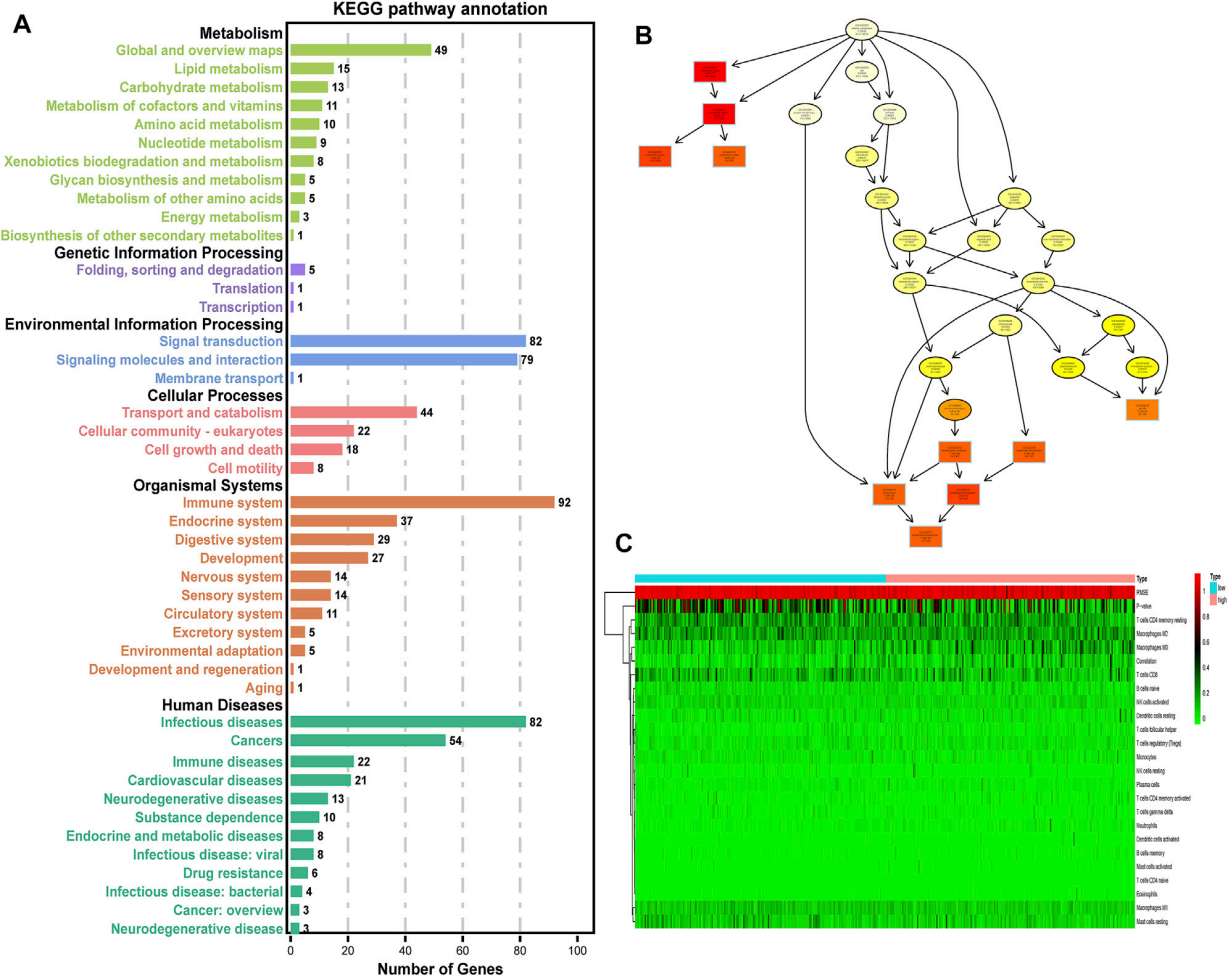
Selection of immune-related processes. **(A)** KEGG enrichment analysis of DEGs between high- and low-risk groups (*p* < 0.05, *q* < 0.05). **(B)** Cellular component pathway of GO analysis. **(C)** Heatmap of 22 tumor immune-infiltrated cells for each HCC patient in TCGA cohort. Low: low-risk group of the ERG signature. High: high-risk group of the ERG signature.

The differential genes between low- and high-risk groups were engaged in both intercellular and extracellular processes (Figure 4B), indicating that our ERG signature may play multi-roles in the cell-to-cell interaction process. The results of the KEGG analysis for differential genes suggested that the immune system had the most enrichment of pathway annotation (Figure 4A). Detailed pathway annotation results are shown in Supplementary Table S5. Then, 22 tumor immune-infiltrated cells were calculated for each HCC patient in TCGA cohort (Figure 4C). In addition, our analysis further confirmed that the processes related to PI3K-Akt, B cell, NK cell, and CD8^+^ T cell were identified as the best prognostic relevance in all immune system pathways, which was used for further analysis.

### Construction and verification of the RF signature

To more accurately predict HCC prognosis, we constructed an RF model via a nested cross-validation approach. We expected features selected for the final iteration of the RF signature to have higher feature importance for response compared with randomly generated numbers. Before selecting the final set of input features, we normalized each immune-related gene set by univariate Cox regression and LASSO analyses. Only the genes that were significantly associated with the patients’ prognosis could be included (Supplementary Table S2). Finally, we integrated the ERG score (also named as exosome score), the mitosis score, the PI3K-Akt score, the B-cell score, the NK-cell score, and the CD8^+^ T-cell score to establish a new RF signature.

**FIGURE 5:**
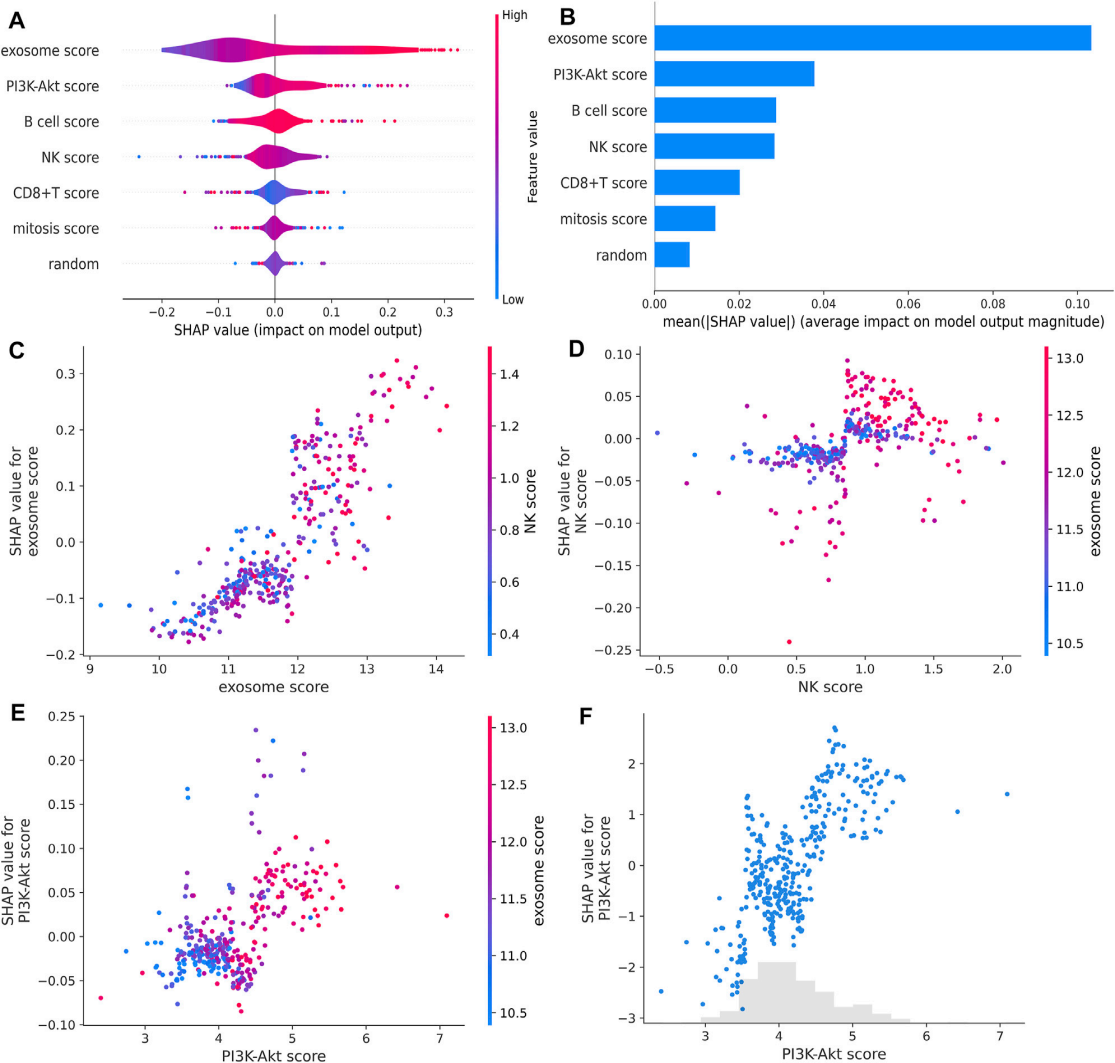
RF feature importance and interactions. **(A,B)** RF feature importance compared with randomly generated numbers estimated with SHAP. **(C,D)** Impact of the interaction between the exosome score and NK score estimated with SHAP. **(E)** Impact of the interaction between the PI3K-Akt score and exosome score estimated with SHAP. **(F)** PI3K-Akt score for each patient in TCGA estimated with SHAP.

**FIGURE 6:**
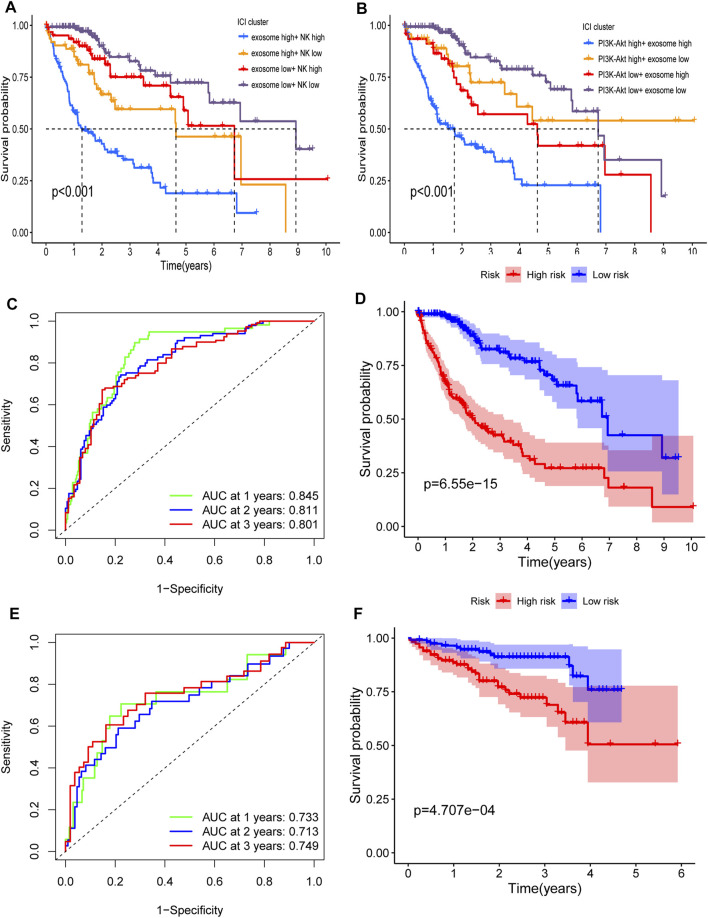
Interaction and verification of the RF signature. **(A)** Interaction of the exosome score and NK score. Kaplan–Meier curves for the OS of patients in exosome score high + NK score high, exosome score high + NK score low, exosome score low + NK score high, and exosome score low + NK score low in TCGA cohort (*p* <0.001). **(B)** Interaction of the PI3K-Akt score and exosome score. Kaplan–Meier curves for the OS of patients in PI3K-Akt score high + exosome score high, PI3K-Akt score high + exosome score low, PI3K-Akt score low + exosome score high, and PI3K-Akt score low + exosome score low in TCGA cohort (*p* <0.001). **(C,D)** AUC of time-dependent ROC curves **(C)** and Kaplan–Meier curves **(D)** of the RF signature in TCGA. **(E,F)** AUC of time-dependent ROC curves **(E)** and Kaplan–Meier curves **(F)** of the RF signature in ICGC.

Next, we compared the contribution of our input feature with randomly generated numbers. Notably, each RF feature had higher feature importance than randomly generated numbers, and the exosome score had the most significant association with response (Figures 5A,B). We also identified the interactions between the variables. We found that the high expression of the exosome score combined with the high expression of NK score had a better predictive performance (Figures 5C,D). These data suggest that our RF signature has the best prognostic accuracy for those HCC patients who have both high exosome scores and high NK scores among all the groups (Figure 6A). Furthermore, the patients with both high-risk exosome scores and high-risk NK scores had the worst survival prognosis. Moreover, we found an association between the high expression of exosome score and high expression of PI3K-Akt score (Figure 5E), indicating that the RF signature has the best predictive performance for those patients who have both high-risk PI3K-Akt scores and high-risk exosome scores. Furthermore, the patients with both high-risk PI3K-Akt scores and high-risk exosome scores also had the worst survival prognosis among all the groups (Figure 6B). Additionally, the value of the PI3K-Akt score is shown in Figure 5F.

The RF signature showed a better predictive performance than the ERG signature (Figure 6D, *p* = 6.55 × 10^−15^), with an AUC of 0.845 for 1 year, 0.811 for 2 years, and 0.801 for 3 years (Figure 6C) in TCGA cohort. In the ICGC cohort, the RF signature also had a better performance (Figure 6F, *p* = 4.707 × 10^−4^), with an AUC of 0.733 for 1 year, 0.713 for 2 years, and 0.749 for 3 years (Figure 6E).

### Validation of the expression of prognostic genes by quantitative real-time PCR and IHC

**FIGURE 7:**
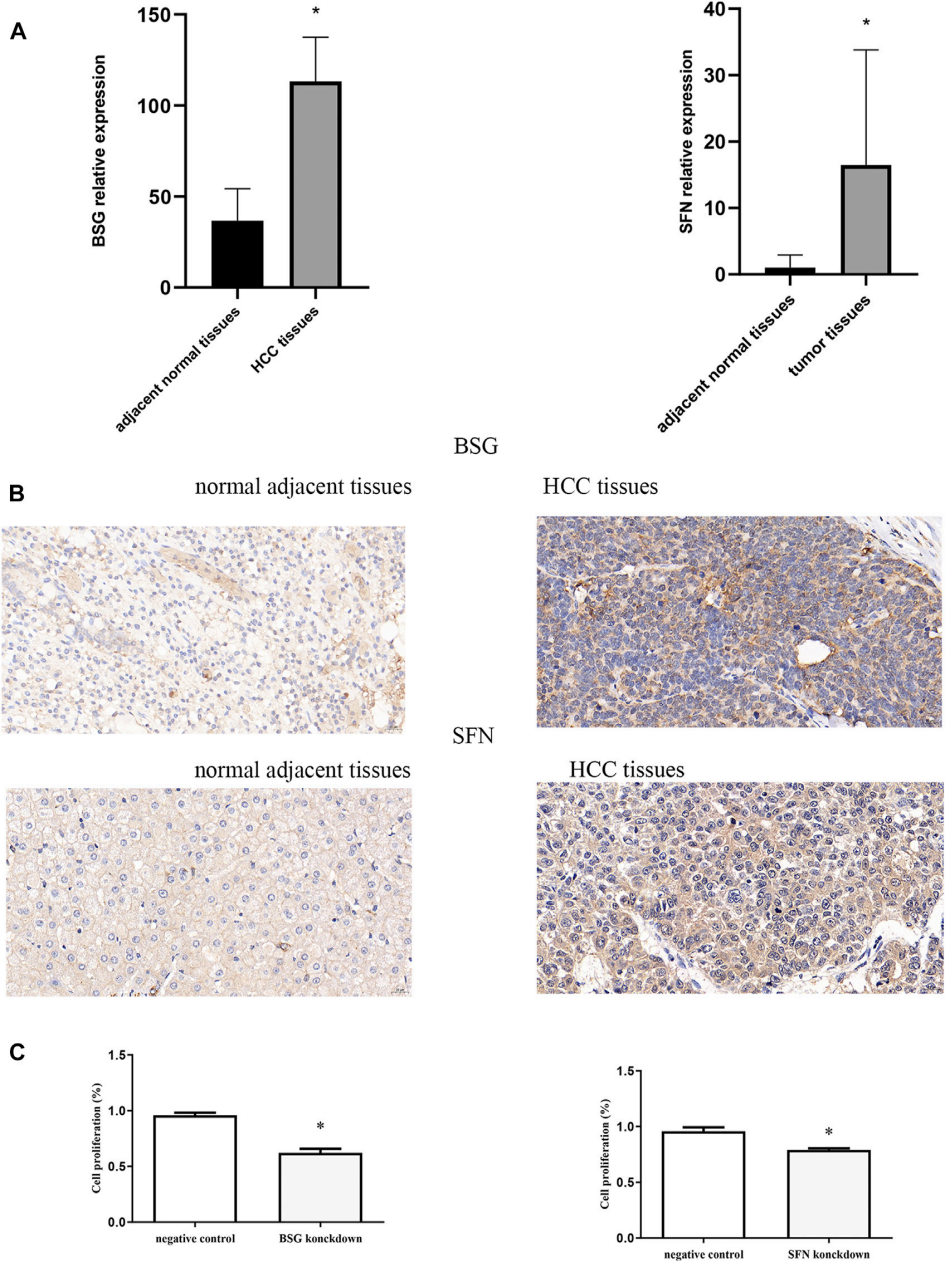
Quantitative real-time PCR, immunohistochemistry, and CCK-8. **(A)** mRNA expression of BSG and SFN. **(B)** Levels of BSG and SFN were analyzed by IHC. The scale bar represents 20 μm. **(C)** Cell proliferation *p<0.05.

Whether these prognostic genes are dysregulated in HCC was further examined. BSG and SFN, members of 13 ERGs, were examined in HCC tissues in an independent sample cohort via quantitative real-time PCR and IHC. We found that BSG and SFN were highly expressed in HCC samples compared with adjacent normal tissues (Figures 7A,B). In line with it, similar results were shown in IHC, which were consistent with the mRNA results of BSG and SFN in TCGA and ICGC.

### Effects of inhibition of BSG and SFN on cell proliferation

The effects of inhibition of BSG and SFN on cell proliferation were next examined using CCK-8. Our results showed that the knockdown of BSG as well as SFN inhibition led to a significant reduction in cell proliferation (Figure 7C).

## Discussion

It has been reported that tumor cells have a higher mitotic frequency than normal cells (Tao, 2005). Mitosis is confirmed as an anti-tumor target. In fact, drugs that stop cells in mitosis, also called anti-mitoses, are common treatments for a variety of human cancers, including breast, non-small-cell lung, and ovarian cancers (Weaver and Cleveland, 2005). Several studies have reported that exosomes can affect the mitosis of tumor cells. For example, Oliver et al. found that exosomes derived from human primed mesenchymal stem cells induce tumor cell mitosis (Yuan et al., 2019), and similar results could be found in colon cancer cells (Ren et al., 2019). In addition, exosomes produced by tumor cells have been demonstrated to play an important role in immune regulation. Exosomes have been demonstrated to promote inflammation, infectious diseases, and autoimmunity *via* regulating immune stimulation or suppression (Robbins and Morelli, 2014). All aforementioned evidence suggests that a combination of exosome, mitosis, and immunity may have potential predictive values for HCC.

In this study, we used two machine learning methods (univariate feature selection and RF algorithm) to screen out 13 ERGs, which were used to construct an ERG signature. Finally, this ERG signature was demonstrated to have a significant prognostic prediction performance. Then, enrichment analysis identified some remarkable factors that correlated with the prognosis of HCC patients. We found that patients with high-risk exosome scores and high-risk NK scores had the worst survival. Subsequently, ERG, mitosis, PI3K-Akt pathway, B cell, NK cell, and CD8^+^ T cell were integrated into an RF signature via RF algorithm. This RF signature was demonstrated to have a better prognostic prediction performance than any single factor. In HCC tissues, we also assessed the dysregulation of BSG and SFN, members of key prognostic genes of HCC. Clearly, it was found that BSG and SFN were enhanced in HCC tissues. Inhibition of BSG or SFN led to a reduction in HCC proliferation.

In fact, many prognostic and predictive signatures for tumor patients based on exosome-related processes have been developed (Wu et al., 2021; Li et al., 2022; Lin et al., 2022; Wu et al., 2022). But, taking only exosome-related processes into consideration may not be enough. Recently, it has been reported that immune-related responses are also included in tumor prognostic signatures. Combined with these, an effective predictive model should take different components of the immune system into consideration, and its predictive response may change according to different cancer types (Bruni et al., 2020). Increasing evidence has shown the involvement of B cells (Sautes-Fridman et al., 2019; Cabrita et al., 2020; Helmink et al., 2020; Petitprez et al., 2020; Sharonov et al., 2020), NK cells (Andre et al., 2018), and CD8^+^ T cells (Mariathasan et al., 2018) in cancer immunotherapy responses and immunosurveillance. In our study, we combined B cell, NK cell, and CD8^+^ T cell parameters with the tumor microenvironment, exosome, mitosis, and PI3K-Akt pathway. In addition, to the best of our knowledge, this is the first report to use a novel machine learning method (RF algorithm) to construct this signature.

However, this study still has some disadvantages. For example, our RF signature was only validated in TCGA and ICGC datasets, and more external validation sets should be included. In addition, exosome-, mitosis-, and immune-related processes, which are used to construct the RF model, should have more experimental validation to elaborate the relationships among them.

In conclusion, we constructed an RF signature that contained exosome, mitosis, PI3K-Akt, B cell, NK cell, and CD8^+^ T cell signaling. The RF signature can accurately predict the prognosis of HCC patients and has potential clinical value.

## Data Availability

The datasets presented in this study can be found in online repositories. The names of the repository/repositories and accession numbers can be found in the article/Supplementary Material.
