# Supplementary figures and images for: Machine learning identifies exosome features related to hepatocellular carcinoma
Source: Front Cell Dev Biol. 2022 Sep 19;10:1020415. doi: 10.3389/fcell.2022.1020415 (PMC9527306; doi:10.3389/fcell.2022.1020415)

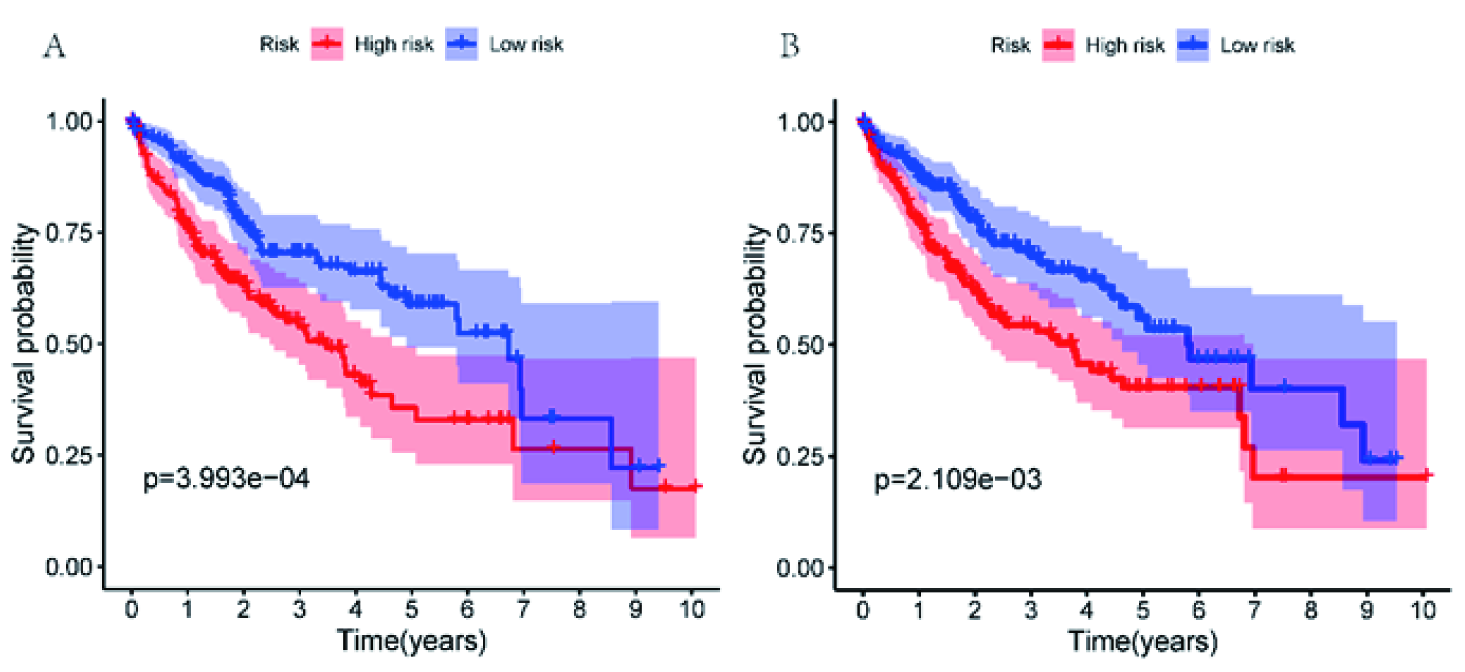

Supplement: Supplementary file 4 [file Image1.TIF]
